# QTL associated with resistance to defoliation *(Cylindrocladium pteridis)* in *Eucalyptus spp.*

**DOI:** 10.1186/1753-6561-5-S7-P27

**Published:** 2011-09-13

**Authors:** Talyta G Zarpelon, Lúcio MS Guimarães, Marcelo M Coutinho, Braz Cápua Neto, Danielle A Faria, Dario Grattapaglia, Acelino C Alfenas

**Affiliations:** 1 Universidade Federal de Viçosa, Brazil; 2 Clonar Resistência a Doenças Florestais, Brazil; 3 Embrapa Cenargen, Brazil

## 

Leaf blight and defoliation caused by *Cylindrocladium pteridis* is one of the main leaf diseases found in Brazilian eucalypt plantations in warm and high rainfall regions. The inter and intraspecific variability in the intensity of defoliation indicate that your control can be achieved by planting resistant genotypes. In order to guide breeding programs and to predict the stability of resistance is essential to understand the genetic control of resistance in eucalypts to defoliation. Thus, the objectives of this paper were to map QTLs (Quantitative Trait Loci) associated with resistance to defoliation in a *Eucalyptus urophylla* x *E. camaldulensis* (EU11 x EC06) family and validate microsatellite markers linked to QTLs in four unrelated pedigrees involving different *Eucalyptus* species. Defoliation was evaluated on the branches of the lower third of the plants inoculated under controlled conditions. We identified five QTLs for resistance to defoliation (Rd), three on the map of the parental EU11, Rd-1 and Rd-2 located on linkage group (LG) 1 and Rd-4 located on LG6; and two QTLs located on the map of the parental EC06, present in LG2 (Rd-3) and LG8 (Rd-5). Markers linked to QTLRd-2 were validated in two unrelated families by simple linear regression analysis. This is the first genomic map of QTLs associated with resistance to defoliation caused by *C. pteridis* in *Eucalyptus*.

